# Getting the guts to expand stroke treatment: The potential for microbiome targeted therapies

**DOI:** 10.1111/cns.13988

**Published:** 2022-10-10

**Authors:** Molly Monsour, Davide M. Croci, Siviero Agazzi, Cesario V. Borlongan

**Affiliations:** ^1^ University of South Florida Morsani College of Medicine Tampa Florida USA; ^2^ Department of Neurosurgery and Brain Repair University of South Florida, Morsani College of Medicine Tampa Florida USA; ^3^ Center of Excellence for Aging and Brain Repair University of South Florida Morsani College of Medicine Tampa Florida USA

**Keywords:** gut‐brain axis, hemorrhagic transformation, ischemic stroke, microbiota, stem cells

## Abstract

**Aims:**

This review focuses on the recent literature regarding the role of the gut‐brain axis (GBA) following ischemic stroke.

**Discussion:**

Stroke is the 5th leading cause of death and disability in the United States; however, few therapies have been developed to improve prognoses. There is a plethora of evidence suggesting peripheral inflammatory responses play a large role in the pathogenesis of stroke. Additionally, hyperglycemic conditions may play a significant role in worsening stroke outcomes due to microbiome dysbiosis.

**Conclusion:**

Recent research has illuminated the vital role of the GBA in propagating poor clinical outcomes, such as hemorrhagic transformation, following ischemic stroke. Considering this detrimental consequence of stroke, and the apparent role of the GBA role, future therapeutics should aim to mitigate this peripheral contribution to stroke complications.

## Graphical abstract

This review highlights microbiome dysbiosis in stroke, specifically how hyperglycemic conditions may contribute to disastrous complications. Treatments focused on ameliorating the stroke‐induced pro‐inflammatory gut‐brain axis communication are discussed.

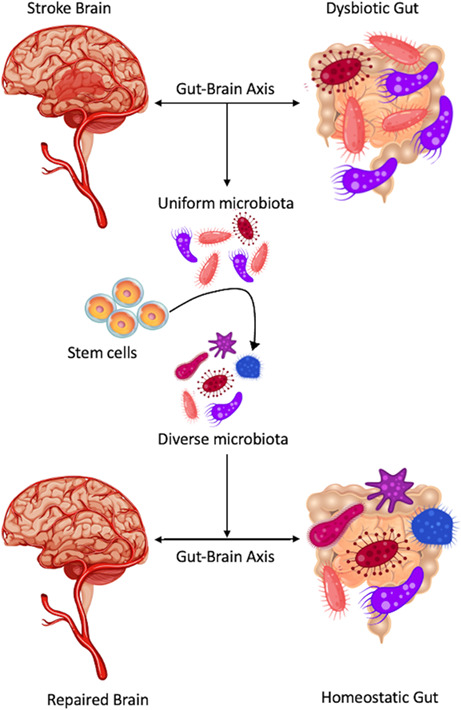

## 1 CENTRAL AND PERIPHERAL INFLAMMATORY RESPONSES AFTER STROKE

Ischemic stroke refers to the loss of blood flow to an area of the brain, commonly due to vascular disease induced oxygen and nutrient deprivation.^1^ The impact of ischemic stroke on the population is momentous, killing nearly 130,000 people annually in the United States and having a mortality rate of 25%.^2^, ^3^, ^4^ Stroke also results in extreme functional deficits, ranked as the number one cause of long‐term disability.^5^ Unfortunately, few treatments are available for stroke. Front‐line treatments currently include tissue plasminogen activator (tPA) and mechanical thrombectomy (MT). Both therapeutics, however, must be employed within a short time window, and have many associated risks, such as negatively shifting immune responses to stroke and hemorrhagic transformation (HT).^6^, ^7^, ^8^, ^9^, ^10^ Current research to improve available therapies have focused on the peripheral impacts of stroke and the secondary cell death mechanisms, such as excitotoxicity, oxidative stress, free radical accumulation, mitochondrial dysfunction, impaired neurogenesis, angiogenesis, vasculogenesis, and inflammation.^11^, ^12^, ^13^ Notable peripheral contributors to stroke‐induced neuroinflammation include the spleen, cervical lymph nodes, thymus, bone marrow, gastrointestinal (GI) system, and adrenal glands.^11^, ^14^, ^15^ In particular, the GI system contributes to this systemic inflammatory response via alteration of the microbiome.^16^. Significant research initiatives are ongoing to better understand the bidirectional communication between the gut and brain, termed the gut‐brain axis (GBA). This review highlights recent scientific research advances in this central and peripheral inflammatory responses associated with stroke pathology and its treatment.

## 2 IS IT BUTTERFLIES OR MICROBIOTA?

The gut contains enormous immune cell reserve within its Peyer's patches, lamina propria immune cells, intraepithelial lymphocytes, and mesenteric lymph nodes,^14^ accounting for more than 70% of the body's immune system.^17^ When in need of an immune response, the CNS sends parasympathetic and sympathetic signals to the gut, ultimately regulating gut motility, secretions, permeability, microbiota, and immune cell activity.^18^ Following these signals, the gut responds by sending vagal signals and microbiota signals, such as endotoxins, neurotransmitters, short chain fatty acids, indoles, or bile acids.^18^ This dynamic communication has been deemed the gut‐brain axis (GBA),^19^ and has been hypothesized to play a dominant role in many neurological pathologies, such as Alzheimer's disease, general cognitive decline, stroke, emotional dysregulation, and memory.^20^, ^21^, ^22^ As a result of stroke‐induced sympathetic signaling, decreased microbiota diversity, altered microbiota populations, and inflammatory T‐cell infiltration are noted within the gut and correlate with poor stroke outcomes, such as hemorrhagic transformation (HT).^23^, ^24^, ^25^, ^26^

HT is a deadly outcome of ischemic stroke possibly related to oxidative stress, inflammation, hyperglycemia, and other blood brain barrier (BBB) weakening factors.^11^, ^12^, ^13^, ^27^ The functioning BBB prohibits extracerebral components from entering the CNS, such as immune cells and pro‐inflammatory signals.^28^, ^29^ After ischemic incidences, however, damage‐associated molecular proteins (DAMPs), cytokines, and chemokines related to stroke can escape the CNS, activate systemic inflammation, and recruit peripheral macrophages, neutrophils, and lymphocytes to surpass the weakened BBB.^30^ Recently, studies have proposed that the gut is a major contributor to bidirectional inflammatory signaling in stroke. Yet another critical contribution of the gut to stroke pathology includes its role in HT.^31^ This review focuses on the microbiota changes observed after stroke, along with the role of glucose in modulating GBA‐induced HT.

## 3 GUT‐WRENCHING CONSEQUENCES OF THE GBA

Understanding the role of the gut microbiota in stroke pathophysiology, accumulating evidence has aimed to determine whether the GBA can influence the deadly stroke consequence of HT. A major hypothesized contributor to altered GBA communications prompting HT involves glucose metabolism. Glucose has been shown to increase bacteria capable of breaking down the guts' mucus barrier, ultimately increasing inflammatory contributions.^32^ Thus, HG rat models can aid in analyzing the role of glucose in microbiota makeup and stroke outcomes.^31^ This is a novel and innovative idea, especially considering the high risk for stroke in patients with diabetes.^33^, ^34^ After undergoing middle cerebral artery occlusion, HG rats show worse outcomes, greater infarct volumes, and greater mortality compared to normoglycemic (NG) rats.^31^ Since a large amount of glucose absorption occurs in the gut, facilitated by microbiota,^35^ it is logical that HG models offer a great deal of insight to microbiota changes noted in stroke patients.

### 3.1 Hyperglycemic stroke induces inflammatory microbiome shifts

HG models demonstrate shifts in inflammatory microbiota composition.^31^ In total, HG rats show 35 variations (from phylum to genus levels) from NG rats and 30 taxa are notably increased in abundancy compared to control rats. These include a plethora of pro‐inflammatory taxons, such as *Actinobacteria*, *Proteobacteria*, *Verrucomicrobia*, *Synergistetes*, *Erysipelotrichaceae*, *Butyricimonas*, and *Desulfovibrio*.^36^ In depth microbiota analysis revels significantly higher *Firmicutes/Bacteroidetes* (F/B) in NG rats compared to control groups. HG rats show even higher F/B levels, differing significantly from the NG rats. HG rats also show elevations in anaerobic *Proteobacteria* and *Actinobacteria,* ^31^ suggesting that hyperglycemia may promote anaerobic proliferation by shutting down oxygen formation in the gut.^37^ Importantly, the phylum *Proteobacteria* includes *Enterobacteriaceae*, which have been shown to amplify inflammation after infarction.^25^ In a mouse model of stroke, *Enterobacteriaceae* expansion was related to brain and gut ischemia induced nitrate formation. Systemic inflammation due to microbiota signals were amplified, worsening stroke outcomes.^25^ Furthermore, F/B and *Actinobacteria* are also pro‐inflammatory and known to proliferate in stroke patients.^38^ This inflammation induced by dysbiosis may contribute to HT after stroke by weakening the BBB.

**FIGURE 1:**
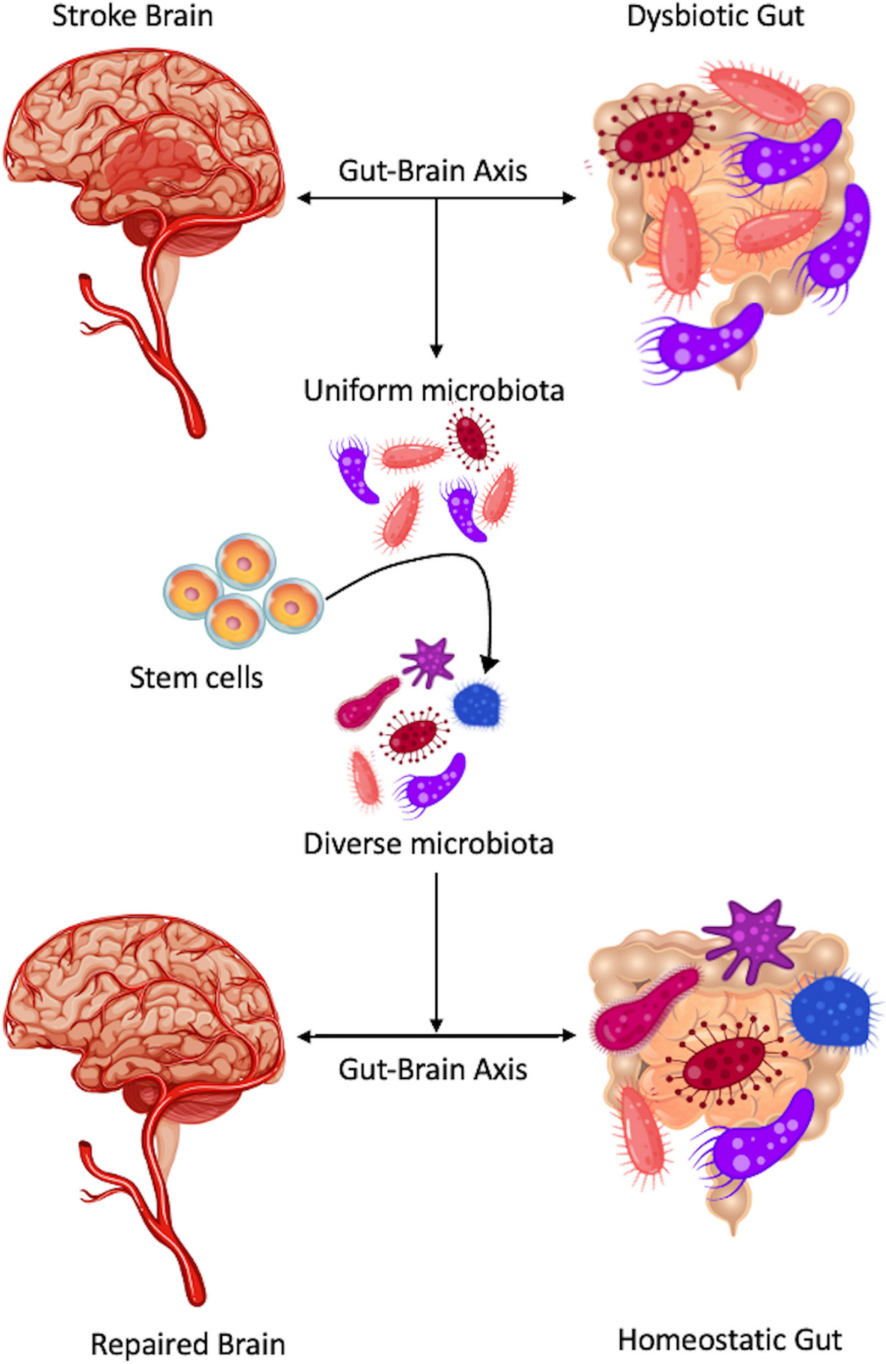
This figure exemplifies the impact of stroke on altering the gut microbiome, which ultimately impacts the CNS and stroke pathology. Potential treatment with stem cells may mitigate these deleterious effects

The GBA communicates largely through inflammatory mediators, which may be novel targets for altering GBA contributions to stroke pathogenesis.^16^ Aberrant signals from the gut after stroke utilize BBB disruption to relay information to the brain. MMP‐9 contributes to BBB weakening^39^ and is higher in rats with HT.^31^ Higher MMP‐9 correlates with lower SFCA, propanoic acid concentration, *Holdemania* and *Collinsella* and correlates with higher *Allobaculum*, *Erysipelotrichi*, *Erysipelotrichales*, and *Erysipelotrichaceae*.^31^ Notably, *Holdemania* induces anxiety, neuroinflammation, and negatively impacts metabolism.^40^, ^41^ In addition to contributing to BBB permeability, inflammatory cytokines TNF‐α, IL‐17, and IL‐1β are higher in HG and NG rats compared to controls. These cytokines are also significantly more elevated in HG rats compared to NG rats. IL‐10, an anti‐inflammatory cytokine is lower in HG and NG rats compared to controls. This inflammatory phenotype is also related to microbiota composition after stroke in murine models, with c_Erysipelotrichi, o_Erysipelotrichales, and f_Erysipelotrichaceae positively correlated to high levels of inflammatory cytokines. Contrarily, g_Holdemania, g_Collinsella, f_Lachnospiraceae, and g_Blautia negatively correlate to increases in inflammatory cytokines. Elevated g_Escherichia and p_proteobacteria directly relate to increased TNF‐α levels in stroke models. Regarding SFCAs, lower acetic acid and propionic acid are associated with higher IL‐17 levels. In addition to altered inflammatory signaling, microbiota composition shifts also result in abnormal metabolic pathways, with HG groups showing greater carbohydrate metabolism, energy production, and energy conversion compared to NG.^31^ There is an apparent relationship between the microbiota composition, inflammation, and BBB permeability, solidifying the role of the GBA in stroke pathology (Figure 1).

### 3.2 Altered mucosal membranes in the setting of hyperglycemic stroke

HG may also dampen mucosal barriers by increasing proliferation of mucolytic bacteria. Mucolytic bacteria, such as *Prevotella*, *Akkermansia*, *Streptococcus*, and *Helicobacter* are increased in HG rats, suggesting a mechanism for GBA dysregulation.^31^, ^32^, ^36^, ^42^ Glucose has been shown to weaken the guts' mucosal barrier,^32^ and permeable gut barriers can be seen in NG rat models of stroke as well.^36^ Thus, a combination of HG and stroke may exacerbate the inflammatory GBA communication via increased barrier permeability and facilitated communication. Freed microbiota can enter the body and cause several detrimental consequences, which worsen stroke outcome, such as endotoxemia and infection.^36^, ^43^ Subsequent immune reactions may further amplify the body's inflammatory responses to stroke, weakening the BBB further, and increasing the risk of HT and poor prognosis.

### 3.3 Sugars or fats: hyperglycemic stroke alters SCFAs

While the microbiome is significantly different among HG rats, the SCFA makeup may also impact stroke outcomes.^44^ Of note, utyric acid, propanoic acid, isobutyric acid, isovaleric acid, valeric acid, and hexanoic acid are decreased in HG rats compared to controls.^31^ Valeric acid levels are decreased in HG rat feces compared to NG rat feces. Ultimately, HG rat feces have lower SCFA levels compared to control and NG rat feces, despite increased *Ruminococcaceae* and *Lachnospiraceae* in HG rats, both of which largely contribute to SCFA levels.^31^ SCFA have been shown to improve stroke outcomes, as some SCFAs can induce neurogenesis and decrease inflammation.^45^, ^46^ Administration of SCFAs in mice after stroke alter microglial activation to enhance structural and functional remodeling.^45^ Contrarily, reduction of SCFAs, such as butyrate, may detriment stroke outcomes, as shown in HG rats.^31^ Butyrate is a key SCFA in energizing intestinal epithelial cells, regulating immune reactions, and modulating metabolic processes.^47^, ^48^ Lack of butyrate producing bacteria correlates with increased stroke risk, type II diabetes, obesity, and cardiovascular disease.^49^, ^50^, ^51^, ^52^ Lower butyrate producing bacteria are observed in stroke patients and correlate to higher infection rates following stroke.^44^ Thus, replacing SFCA directly or via microbiome renewal may mitigate stroke damage. The wide range of microbiome changes suggests novel therapeutic targets within the GBA for stroke.

## 4 MANAGING THE MICROBIOME

Given the significant role of the GBA in stroke pathogenesis and prognosis, therapies should target this injurious dysbiosis. As noted, Enterobacteriaceae contribute to ischemic complications through LPS reactions with macrophages, ultimately inducing free radical formation.^25^ To ameliorate this effect, aminoguanidine, superoxide dismutase, and tungstate treatments can be employed to lessen nitrate formation. More directly, Enterobacteriaceae concentrations can be reduced.^25^ More drastic approaches to normalizing the microbiome involve recolonizing the gut to alter immune responses to stroke. In a germ‐free rat model, fecal samples were taken from HG rats, which underwent HT. Microbiota transplantations from these samples were given to germ‐free mice. MCAO was conducted 2 weeks later. Germ‐free rats show improved infarct volumes, rates of HT and hemoglobin levels, inflammatory cytokine and MMP concentrations, and functionality compared to control rats.^31^ Other results support these findings, showing that fecal transplants are beneficial in adapting a healthy microbiome following stroke.^24^ In a similar model of germ‐free mice undergoing stroke, amoxicillin can increase anti‐inflammatory regulatory T‐cell populations while decreasing pro‐inflammatory Th‐17 infiltration in the gut. Amoxicillin treatment demonstrates reduced infarct volumes and functional deficits.^53^ Other methods targeting Treg differentiation from microbiome activation may be beneficial as well, such as IL‐2/IL‐2R antibody complexes.^54^ Normalizing the microbiome may be a novel direction for research initiatives in regulating GBA communications following stroke.

Other potential therapeutic targets may involve microbiome products, such as TMAO or SFCA. Elevated TMAO is related to atherosclerosis by prompting foam cell formation, however, following stroke, levels of TMAO are decreased alongside microbiota shifts.^38^, ^55^, ^56^ It is questionable whether down or up regulation of TMAO would be the most beneficial for stroke therapeutics.^38^ Butyrate, previously discussed due to its neuroprotective properties, also demonstrates reduced infarct volumes and improved sensory motor function in MCAO rat models.^57^ These effects may be due to the direct replenishment of butyrate, which is decreased after stroke.^44^ As noted previously, SFCA administration after stroke in mice can improve motor function and enhanced neural connectivity via altered inflammatory signaling.^45^, ^46^ Restoring homeostatic levels of TMAO and SCFA can ameliorate harmful microbiome induced side effects, and potentially lessen aberrant inflammatory signals via the GBA.

Interventions targeting GBA permeability and communication capabilities may be beneficial, for example, targeting cytokines or MMPs.^58^, ^59^ Human bone marrow‐derived NCS‐01 cells offer therapeutic effects for ischemic stroke, likely by secreting fibroblast growth factor and IL‐6. IL‐6 is unique in its double‐edged contributions to inflammation, acting as a pro‐ and anti‐inflammatory cytokines.^60^ While stem cell studies have shown increased IL‐6 production mitigate stroke damage,^59^ pharmaceutical interventions show the opposite.^61^ While direct intervention in cytokine processing via stem cells may be beneficial in targeting the GBA, further research is needed to determine whether IL‐6 acts as a pro‐ or anti‐inflammatory mediator in GBA signaling. Combination therapy including Puerariae Lobatae Radix (PLR) and Chuanxiong Rhizoma (CXR), traditional Chinese medicines, have also been shown to lessen stroke damage by relieving dysbiosis and protecting the brain‐gut mucosal barriers and preventing detrimental GBA communications.^36^, ^62^, ^63^ Given the recent interest in the GBA and its role in stroke outcomes,^64^ further research is needed to establish treatments targeting this bimodal inflammatory signaling and resultant dysbiosis.

Stem cells may also be a novel intervention to intercede the GBA signaling. Stem cells can directly impact stroke outcomes when targeting neural inflammation^11^, ^65^; however, there is also some evidence suggesting stem cells exert a bystander effect by ameliorating peripheral inflammation.^66^ In fact, bone marrow mesenchymal cells can modulate dysbiosis, while also reducing neuronal death and functional deficits following stroke.^67^ Furthermore, in Parkinson's disease, stem cells have been visualized traveling to the gut to lessen CNS and gut inflammation.^68^ Thus, while not strongly established in the literature, future studies may discover a grand role for stem cells in improving stroke prognosis via interaction with the GBA.

Avoiding medical interventions, simple lifestyle adjustments may also be ideal microbiome normalizers. Ketogenic diets and vegetarian diets show reduced stroke damage and lessen dysbiosis, ultimately improving prognosis.^69^, ^70^ Dietary supplements, such as pro‐ or prebiotics can also reduce stroke severity and lessen the risk of further cardiovascular health crises.^70^ Moreover, regular exercise diminishes stroke‐related microbiome dysregulation, serving as a protective and low maintenance lifestyle adjustment.^71^ Aerobic exercise paired with intermittent fasting can further improve microbiota composition, as measured by organic acid concentrations in feces.^72^ In obese rats, intermittent fasting alone also ameliorates gut dysbiosis and lowers blood high‐density lipoprotein and low‐density lipoprotein. Considering the correlations between obesity and stroke,^73^ intermittent fasting may be another intriguing stroke therapeutic targeting the microbiome.^74^ Acupuncture has also demonstrated anti‐inflammatory powers in neuroinflammatory and inflammatory gut diseases, such as Parkinson's disease and irritable bowel syndrome, by modulating vagal signals to the immune system and subsequent modulation of the GBA.^40^, ^75^, ^76^ Thus, acupuncture may represent a more natural treatment consideration for stroke.^31^ While there are a few potential pharmaceuticals or novel cell‐based therapies for modulating GBA signaling, lifestyle adjustments may be the safest and most feasible approach for improving stroke prognosis by targeting the gut's contributions to peripheral inflammation.

## 5 FOLLOW YOUR HEAD, HEART… AND GUT

These studies gracefully demonstrate the profound role of the microbiome in modulating stroke pathogenesis. Researchers should aim to target stroke‐induced dysbiosis to mitigate peripheral inflammation and, ultimately, improve stroke outcomes. Importantly, the role of HG in the GBA following stroke remains poorly understood but is highly clinically relevant due to the relationship between stroke, diet, and microbiome dysbiosis in HG stroke models.^31^ Considering the brain's protective BBB, the gut may be a more feasible target for stroke therapeutics. Furthermore, the established communicative signaling between these two systems suggests promising discoveries will arise from further analysis of the GBA in stroke.

## AUTHOR CONTRIBUTIONS

M.M. and C.V.B. wrote the main manuscript. M.M. and C.V.B. designed the figure. S.A. and D.C. revised and edited the manuscript.

## CONFLICT OF INTEREST

C.V.B. was funded by the National Institutes of Health (NIH) R01NS090962, NIH R01NS102395, and NIH R21NS109575. Additionally, C.V.B. was funded and received royalties and stock options from Astellas, Asterias, Sanbio, Athersys, KMPHC, and International Stem Cell Corporation and has also received consultant compensation from Chiesi Farmaceutici. C.V.B. also declares patents and patent applications related to stem cell therapy.

## Data Availability

No data availability statement is applicable.

## References

[1] Feigin VL , Krishnamurthi R . Stroke is largely preventable across the globe: where to next? Lancet. 2016;388(10046):733‐734. 27431357 10.1016/S0140-6736(16)30679-1

[2] Marcet P , Santos N , Borlongan CV . When friend turns foe: central and peripheral neuroinflammation in central nervous system injury. Neuroimmunol Neuroinflamm. 2017;4:82‐92. 29670933 10.20517/2347-8659.2017.07 PMC5901724

[3] Mozaffarian D , Benjamin EJ , Go AS , et al. Heart disease and stroke statistics‐‐2015 update: a report from the American Heart Association. Circulation. 2015;131(4):e29‐e322. 25520374 10.1161/CIR.0000000000000152

[4] Andersen KK , Olsen TS , Dehlendorff C , Kammersgaard LP . Hemorrhagic and ischemic strokes compared: stroke severity, mortality, and risk factors. Stroke. 2009;40(6):2068‐2072. 19359645 10.1161/STROKEAHA.108.540112

[5] Acosta SA , Tajiri N , de la Pena I , et al. Alpha‐synuclein as a pathological link between chronic traumatic brain injury and Parkinson's disease. J Cell Physiol. 2015;230(5):1024‐1032. 25251017 10.1002/jcp.24830 PMC4328145

[6] Gumbinger C , Reuter B , Stock C , et al. Time to treatment with recombinant tissue plasminogen activator and outcome of stroke in clinical practice: retrospective analysis of hospital quality assurance data with comparison with results from randomised clinical trials. BMJ. 2014;348:g3429. 24879819 10.1136/bmj.g3429 PMC4039388

[7] Kaesmacher J , Kaesmacher M , Maegerlein C , et al. Hemorrhagic transformations after thrombectomy: risk factors and clinical relevance. Cerebrovasc Dis. 2017;43(5–6):294‐304. 28343220 10.1159/000460265

[8] Nguyen H , Lee JY , Sanberg PR , Napoli E , Borlongan CV . Eye Opener in Stroke. Stroke. 2019;50(8):2197‐2206. 31242827 10.1161/STROKEAHA.119.025249 PMC6650274

[9] Lees KR , Bluhmki E , von Kummer R , et al. Time to treatment with intravenous alteplase and outcome in stroke: an updated pooled analysis of ECASS, ATLANTIS, NINDS, and EPITHET trials. Lancet. 2010;375(9727):1695‐1703. 20472172 10.1016/S0140-6736(10)60491-6

[10] Mao L , Li P , Zhu W , et al. Regulatory T cells ameliorate tissue plasminogen activator‐induced brain haemorrhage after stroke. Brain. 2017;140(7):1914‐1931. 28535201 10.1093/brain/awx111 PMC6059175

[11] Anthony S , Cabantan D , Monsour M , Borlongan CV . Neuroinflammation, stem cells, and stroke. Stroke. 2022;53(5):1460‐1472. 35380050 10.1161/STROKEAHA.121.036948 PMC9038685

[12] Zhao S , Li F , Leak RK , Chen J , Hu X . Regulation of neuroinflammation through programed Death‐1/programed death ligand signaling in neurological disorders. Front Cell Neurosci. 2014;8:271. 25232304 10.3389/fncel.2014.00271 PMC4153295

[13] Stonesifer C , Corey S , Ghanekar S , Diamandis Z , Acosta SA , Borlongan CV . Stem cell therapy for abrogating stroke‐induced neuroinflammation and relevant secondary cell death mechanisms. Prog Neurobiol. 2017;158:94‐131. 28743464 10.1016/j.pneurobio.2017.07.004 PMC5671910

[14] Anrather J , Iadecola C . Inflammation and stroke: an overview. Neurotherapeutics. 2016;13(4):661‐670. 27730544 10.1007/s13311-016-0483-x PMC5081118

[15] Miro‐Mur F , Laredo C , Renu A , et al. Adrenal hormones and circulating leukocyte subtypes in stroke patients treated with reperfusion therapy. Brain Behav Immun. 2018;70:346‐353. 29548995 10.1016/j.bbi.2018.03.018

[16] Yamashiro K , Tanaka R , Urabe T , et al. Gut dysbiosis is associated with metabolism and systemic inflammation in patients with ischemic stroke. PLoS One. 2017;12(2):e0171521. 28166278 10.1371/journal.pone.0171521 PMC5293236

[17] Arya AK , Hu B . Brain‐gut axis after stroke. Brain Circ. 2018;4(4):165‐173. 30693343 10.4103/bc.bc_32_18 PMC6329216

[18] Durgan DJ , Lee J , McCullough LD , Bryan RM Jr . Examining the role of the microbiota‐gut‐brain Axis in stroke. Stroke. 2019;50(8):2270‐2277. 31272315 10.1161/STROKEAHA.119.025140 PMC6646086

[19] Wang HX , Wang YP . Gut microbiota‐brain Axis. Chin Med J (Engl). 2016;129(19):2373‐2380. 27647198 10.4103/0366-6999.190667 PMC5040025

[20] Tillisch K , Mayer EA , Gupta A , et al. Brain structure and response to emotional stimuli as related to gut microbial profiles in healthy women. Psychosom Med. 2017;79(8):905‐913. 28661940 10.1097/PSY.0000000000000493 PMC6089374

[21] Liu P , Jia XZ , Chen Y , et al. Gut microbiota interacts with intrinsic brain activity of patients with amnestic mild cognitive impairment. CNS Neurosci Ther. 2021;27(2):163‐173. 32929861 10.1111/cns.13451 PMC7816203

[22] Cho J , Park YJ , Gonzales‐Portillo B , et al. Gut dysbiosis in stroke and its implications on Alzheimer's disease‐like cognitive dysfunction. CNS Neurosci Ther. 2021;27(5):505‐514. 33464726 10.1111/cns.13613 PMC8025625

[23] Houlden A , Goldrick M , Brough D , et al. Brain injury induces specific changes in the caecal microbiota of mice via altered autonomic activity and mucoprotein production. Brain Behav Immun. 2016;57:10‐20. 27060191 10.1016/j.bbi.2016.04.003 PMC5021180

[24] Singh V , Roth S , Llovera G , et al. Microbiota dysbiosis controls the neuroinflammatory response after stroke. J Neurosci. 2016;36(28):7428‐7440. 27413153 10.1523/JNEUROSCI.1114-16.2016 PMC6705544

[25] Xu K , Gao X , Xia G , et al. Rapid gut dysbiosis induced by stroke exacerbates brain infarction in turn. Gut. 2021. doi:10.1136/gutjnl-2020-323263 33558272

[26] Li N , Wang X , Sun C , et al. Change of intestinal microbiota in cerebral ischemic stroke patients. BMC Microbiol. 2019;19(1):191. 31426765 10.1186/s12866-019-1552-1 PMC6700817

[27] Liu L , Wang Z , Wang X , et al. Comparison of two rat models of cerebral ischemia under hyperglycemic conditions. Microsurgery. 2007;27(4):258‐262. 17477421 10.1002/micr.20351

[28] Hawkins RA , O'Kane RL , Simpson IA , Vina JR . Structure of the blood–brain barrier and its role in the transport of amino acids. J Nutr. 2006;136(1 Suppl):218S‐226S. 16365086 10.1093/jn/136.1.218S

[29] Shinozuka K , Dailey T , Tajiri N , et al. Stem cells for neurovascular repair in stroke. J Stem Cell Res Ther. 2013;4(4):12912. 24077523 10.4172/2157-7633.S4-004 PMC3783263

[30] Wang Q , Tang XN , Yenari MA . The inflammatory response in stroke. J Neuroimmunol. 2007;184(1–2):53‐68. 17188755 10.1016/j.jneuroim.2006.11.014 PMC1868538

[31] Huang Q , Di L , Yu F , et al. Alterations in the gut microbiome with hemorrhagic transformation in experimental stroke. CNS Neurosci Ther. 2022;28(1):77‐91. 34591349 10.1111/cns.13736 PMC8673707

[32] Khan S , Waliullah S , Godfrey V , et al. Dietary simple sugars alter microbial ecology in the gut and promote colitis in mice. Sci Transl Med. 2020;12(567):eaay6218. doi:10.1126/scitranslmed.aay6218 33115951

[33] Hankey GJ . Potential new risk factors for ischemic stroke: what is their potential? Stroke. 2006;37(8):2181‐2188. 16809576 10.1161/01.STR.0000229883.72010.e4

[34] McColl BW , Allan SM , Rothwell NJ . Systemic inflammation and stroke: aetiology, pathology and targets for therapy. Biochem Soc Trans. 2007;35(Pt 5):1163‐1165. 17956302 10.1042/BST0351163

[35] Utzschneider KM , Kratz M , Damman CJ , Hullar M . Mechanisms linking the gut microbiome and glucose metabolism. J Clin Endocrinol Metab. 2016;101(4):1445‐1454. 26938201 10.1210/jc.2015-4251 PMC4880177

[36] Chen R , Wu P , Cai Z , et al. Puerariae Lobatae radix with chuanxiong Rhizoma for treatment of cerebral ischemic stroke by remodeling gut microbiota to regulate the brain‐gut barriers. J Nutr Biochem. 2019;65:101‐114. 30710886 10.1016/j.jnutbio.2018.12.004

[37] Zhao Y , Zhou C , Guo X , et al. Exposed to mercury‐induced oxidative stress, changes of intestinal microflora, and association between them in mice. Biol Trace Elem Res. 2021;199(5):1900‐1907. 32734533 10.1007/s12011-020-02300-x

[38] Yin J , Liao SX , He Y , et al. Dysbiosis of gut microbiota with reduced trimethylamine‐N‐oxide level in patients with large‐artery atherosclerotic stroke or transient ischemic attack. J Am Heart Assoc. 2015;4(11):e002699. 26597155 10.1161/JAHA.115.002699 PMC4845212

[39] Liu W , Hendren J , Qin XJ , Shen J , Liu KJ . Normobaric hyperoxia attenuates early blood–brain barrier disruption by inhibiting MMP‐9‐mediated occludin degradation in focal cerebral ischemia. J Neurochem. 2009;108(3):811‐820. 19187098 10.1111/j.1471-4159.2008.05821.x PMC2676213

[40] Jang JH , Yeom MJ , Ahn S , et al. Acupuncture inhibits neuroinflammation and gut microbial dysbiosis in a mouse model of Parkinson's disease. Brain Behav Immun. 2020;89:641‐655. 32827699 10.1016/j.bbi.2020.08.015

[41] Lippert K , Kedenko L , Antonielli L , et al. Gut microbiota dysbiosis associated with glucose metabolism disorders and the metabolic syndrome in older adults. Benef Microbes. 2017;8(4):545‐556. 28701081 10.3920/BM2016.0184

[42] Derrien M , van Passel MW , van de Bovenkamp JH , Schipper RG , de Vos WM , Dekker J . Mucin‐bacterial interactions in the human oral cavity and digestive tract. Gut Microbes. 2010;1(4):254‐268. 21327032 10.4161/gmic.1.4.12778 PMC3023607

[43] Wells JM , Rossi O , Meijerink M , van Baarlen P . Epithelial crosstalk at the microbiota‐mucosal interface. Proc Natl Acad Sci U S A. 2011;108(Suppl. 1):4607‐4614. 20826446 10.1073/pnas.1000092107 PMC3063605

[44] Haak BW , Westendorp WF , van Engelen TSR , et al. Disruptions of anaerobic gut bacteria are associated with stroke and post‐stroke infection: a prospective case–control study. Transl Stroke Res. 2021;12(4):581‐592. 33052545 10.1007/s12975-020-00863-4 PMC8213601

[45] Kim HJ , Leeds P , Chuang DM . The HDAC inhibitor, sodium butyrate, stimulates neurogenesis in the ischemic brain. J Neurochem. 2009;110(4):1226‐1240. 19549282 10.1111/j.1471-4159.2009.06212.x PMC2726719

[46] Sadler R , Cramer JV , Heindl S , et al. Short‐chain fatty acids improve poststroke recovery via immunological mechanisms. J Neurosci. 2020;40(5):1162‐1173. 31889008 10.1523/JNEUROSCI.1359-19.2019 PMC6989004

[47] Nicholson JK , Holmes E , Kinross J , et al. Host‐gut microbiota metabolic interactions. Science. 2012;336(6086):1262‐1267. 22674330 10.1126/science.1223813

[48] Smith PM , Howitt MR , Panikov N , et al. The microbial metabolites, short‐chain fatty acids, regulate colonic Treg cell homeostasis. Science. 2013;341(6145):569‐573. 23828891 10.1126/science.1241165 PMC3807819

[49] Vital M , Karch A , Pieper DH . Colonic butyrate‐producing communities in humans: an overview using omics data. mSystems. 2017;2(6):e00130‐17. 29238752 10.1128/mSystems.00130-17 PMC5715108

[50] Karlsson FH , Fak F , Nookaew I , et al. Symptomatic atherosclerosis is associated with an altered gut metagenome. Nat Commun. 2012;3:1245. 23212374 10.1038/ncomms2266 PMC3538954

[51] Le Chatelier E , Nielsen T , Qin J , et al. Richness of human gut microbiome correlates with metabolic markers. Nature. 2013;500(7464):541‐546. 23985870 10.1038/nature12506

[52] Qin J , Li Y , Cai Z , et al. A metagenome‐wide association study of gut microbiota in type 2 diabetes. Nature. 2012;490(7418):55‐60. 23023125 10.1038/nature11450

[53] Benakis C , Brea D , Caballero S , et al. Commensal microbiota affects ischemic stroke outcome by regulating intestinal gammadelta T cells. Nat Med. 2016;22(5):516‐523. 27019327 10.1038/nm.4068 PMC4860105

[54] Borlongan MC , Kingsbury C , Salazar FE , et al. IL‐2/IL‐2R antibody complex enhances Treg‐induced neuroprotection by dampening TNF‐alpha inflammation in an In vitro stroke model. Neuromolecular Med. 2021;23(4):540‐548. 33830475 10.1007/s12017-021-08656-0 PMC8613084

[55] Koeth RA , Wang Z , Levison BS , et al. Intestinal microbiota metabolism of L‐carnitine, a nutrient in red meat, promotes atherosclerosis. Nat Med. 2013;19(5):576‐585. 23563705 10.1038/nm.3145 PMC3650111

[56] Tang WH , Wang Z , Levison BS , et al. Intestinal microbial metabolism of phosphatidylcholine and cardiovascular risk. N Engl J Med. 2013;368(17):1575‐1584. 23614584 10.1056/NEJMoa1109400 PMC3701945

[57] Park MJ , Sohrabji F . The histone deacetylase inhibitor, sodium butyrate, exhibits neuroprotective effects for ischemic stroke in middle‐aged female rats. J Neuroinflammation. 2016;13(1):300. 27905989 10.1186/s12974-016-0765-6 PMC5131416

[58] Gao D , Zhang X , Jiang X , et al. Resveratrol reduces the elevated level of MMP‐9 induced by cerebral ischemia–reperfusion in mice. Life Sci. 2006;78(22):2564‐2570. 16321402 10.1016/j.lfs.2005.10.030

[59] Kaneko Y , Lee JY , Tajiri N , et al. Translating intracarotid artery transplantation of bone marrow‐derived NCS‐01 cells for ischemic stroke: behavioral and histological readouts and mechanistic insights into stem cell therapy. Stem Cells Transl Med. 2020;9(2):203‐220. 31738023 10.1002/sctm.19-0229 PMC6988762

[60] Monsour M , Croci DM , Agazzi S . The role of IL‐6 in TBI and PTSD, a potential therapeutic target? Clin Neurol Neurosurg. 2022;218:107280. 35567833 10.1016/j.clineuro.2022.107280

[61] Hudobenko J. Ischemic Stroke Damage Is Reduced by Inhibition of IL‐6 Signaling with Tocilizumab: the University of Texas MD Anderson Cancer Center. The Texas Medical Center Library DigitalCommons@TMC; 2018.

[62] Naito T , Kubota K , Shimoda Y , Sato T , Ikeya Y , Okada M . Effects of constituents in a Chinese crude drug, ligustici chuanxiong rhizoma on vasocontraction and blood viscosity. Nat Med. 1995;49:288‐292.

[63] Chen R , Wu P , Cai Z , et al. The combination of Puerariae Lobatae radix and chuanxiong Rhizoma enhanced the absorption and pharmacokinetics of puerarin by modulating the intestinal barrier and influenced gut microbiota. J Nutrit Biochem. 2019;65:101‐114. 30710886 10.1016/j.jnutbio.2018.12.004

[64] Xia GH , You C , Gao XX , et al. Stroke dysbiosis index (SDI) in gut microbiome are associated with brain injury and prognosis of stroke. Front Neurol. 2019;10:397. 31068891 10.3389/fneur.2019.00397 PMC6491752

[65] Kawabori M , Shichinohe H , Kuroda S , Houkin K . Clinical trials of stem cell therapy for cerebral ischemic stroke. Int J Mol Sci. 2020;21(19):7380. 33036265 10.3390/ijms21197380 PMC7582939

[66] Suzuki T , Sato Y , Kushida Y , et al. Intravenously delivered multilineage‐differentiating stress enduring cells dampen excessive glutamate metabolism and microglial activation in experimental perinatal hypoxic ischemic encephalopathy. J Cereb Blood Flow Metab. 2021;41(7):1707‐1720. 33222596 10.1177/0271678X20972656 PMC8217885

[67] Zhao LN , Ma SW , Xiao J , Yang LJ , Xu SX , Zhao L . Bone marrow mesenchymal stem cell therapy regulates gut microbiota to improve post‐stroke neurological function recovery in rats. World J Stem Cells. 2021;13(12):1905‐1917. 35069989 10.4252/wjsc.v13.i12.1905 PMC8727225

[68] Toledo ARL , Monroy GR , Salazar FE , et al. Gut‐brain Axis as a pathological and therapeutic target for neurodegenerative disorders. Int J Mol Sci. 2022;23(3):1184. 35163103 10.3390/ijms23031184 PMC8834995

[69] Gzielo K , Soltys Z , Rajfur Z , Setkowicz ZK . The impact of the ketogenic diet on glial cells morphology. a quantitative morphological analysis. Neuroscience. 2019;413:239‐251. 31220541 10.1016/j.neuroscience.2019.06.009

[70] Zhao L , Yang L , Guo Y , Xiao J , Zhang J , Xu S . New insights into stroke prevention and treatment: gut microbiome. Cell Mol Neurobiol. 2022;42(2):455‐472. 33635417 10.1007/s10571-021-01047-w PMC11441219

[71] Kingsbury C , Shear A , Heyck M , et al. Inflammation‐relevant microbiome signature of the stroke brain, gut, spleen, and thymus and the impact of exercise. J Cereb Blood Flow Metab. 2021;41(12):3200‐3212. 34427146 10.1177/0271678X211039598 PMC8669279

[72] Soares NL , Dorand VAM , Cavalcante HC , et al. Does intermittent fasting associated with aerobic training influence parameters related to the gut‐brain axis of Wistar rats? J Affect Disord. 2021;293:176‐185. 34214787 10.1016/j.jad.2021.06.028

[73] Kernan WN , Inzucchi SE , Sawan C , Macko RF , Furie KL . Obesity: a stubbornly obvious target for stroke prevention. Stroke. 2013;44(1):278‐286. 23111440 10.1161/STROKEAHA.111.639922

[74] Rong ZH , Liang SC , Lu JQ , et al. Effect of intermittent fasting on physiology and gut microbiota in presenium rats. Nan Fang Yi Ke Da Xue Xue Bao. 2016;37(4):423‐430. 28446391 10.3969/j.issn.1673-4254.2017.04.01 PMC6744091

[75] Chey WD , Maneerattaporn M , Saad R . Pharmacologic and complementary and alternative medicine therapies for irritable bowel syndrome. Gut Liver. 2011;5(3):253‐266. 21927652 10.5009/gnl.2011.5.3.253 PMC3166664

[76] Torres‐Rosas R , Yehia G , Pena G , et al. Dopamine mediates vagal modulation of the immune system by electroacupuncture. Nat Med. 2014;20(3):291‐295. 24562381 10.1038/nm.3479 PMC3949155

